# Whole genome DNA methylation sequencing of the chicken retina, cornea and brain

**DOI:** 10.1038/sdata.2017.148

**Published:** 2017-10-10

**Authors:** Isac Lee, Bejan A. Rasoul, Ashton S. Holub, Alannah Lejeune, Raymond A. Enke, Winston Timp

**Affiliations:** 1 Department of Biomedical Engineering, Johns Hopkins University, Baltimore, MD 21218, USA; 2 Department of Biology, James Madison University, Harrisonburg, VA 22807, USA; 3 Center for Genome & Metagenome Studies, James Madison University, Harrisonburg, VA 22807, USA

**Keywords:** DNA methylation, Retina, Organogenesis

## Abstract

Whole genome bisulfite sequencing (WGBS) analysis of DNA methylation uses massively parallel next generation sequencing technology to characterize global epigenetic patterns and fluctuations throughout a range of tissue samples. Development of the vertebrate retina is thought to involve extensive epigenetic reprogramming during embryogenesis. The chicken embryo (*Gallus gallus*) is a classic model system for studying developmental biology and retinogenesis, however, there are currently no publicly available data sets describing the developing chicken retinal methylome. Here we used Illumina WGBS analysis to characterize genome-wide patterns of DNA methylation in the developing chicken retina as well as cornea and brain in an effort to further our understanding of retina-specific epigenetic regulation. These data will be valuable to the vision research community for correlating global changes in DNA methylation to differential gene expression between ocular and neural tissues during critical developmental time points of retinogenesis in the chicken retina.

## Background & Summary

Advances in next generation sequencing (NGS) technology have substantially increased the number of species with completed high quality genome assemblies. These advances have also opened new doors to studying the functionality of complex genomes^1^. The epigenetics community has benefited greatly from NGS experiments mapping genome-wide profiles of specific histone tail and genomic DNA modifications. Consortium projects such as the ENCODE Project and the Roadmap Epigenomics Project have deepened our understanding of numerous specialized human cell and tissue types and have paved the way for similar experiments in model systems of human development and disease^2,3^.

The experiment described here is part of a larger ongoing project within the James Madison University’s Center for Genome and Metagenome Studies (CGEMS) investigating transcriptional regulation in the developing vertebrate retina. The retina, a stratified layer of sensory neurons that lines the posterior portion of the eye, contains rod and cone photoreceptors that absorb focused light photons and convert their energy into electrochemical signals transmitted to the brain and processed into what we perceive as the visual world. Within the developing retina, complex transcriptional networks regulate proper differentiation of specialized subclasses of retinal neurons^4^. Characterizing epigenetic regulation of these transcriptional networks is critical for further understanding molecular mechanisms driving retinal development as well as for crafting novel strategies to combat blinding human diseases that affect the retina.

**Figure 1:**
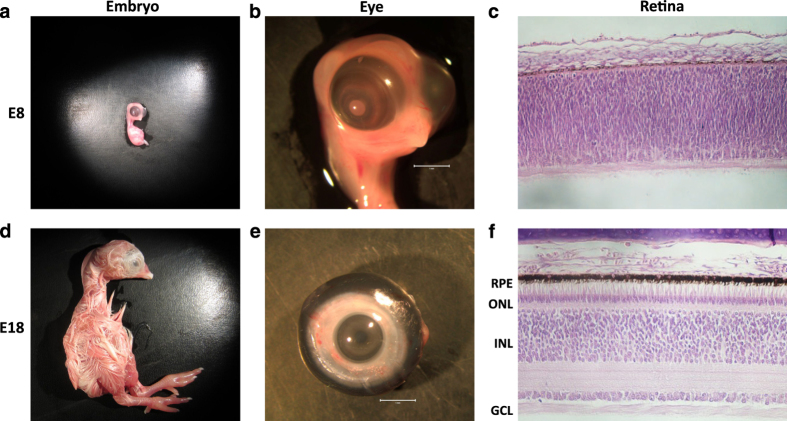
Overview of the chicken embryo, eye, and retinal development. Image of embryonic day 8 (E8) and E18 embryos (**a**,**d**), eyes (**b**,**e**) and H+E stained retinal cross sections (**c**,**f**). Cross section abbreviations: Retinal Pigmented Epithelium (RPE), Outer Nuclear Layer (ONL), Inner Nuclear Layer (INL), and Ganglion Cell Layer (GCL).

The chicken (*Gallus gallus*) embryo is a reliable and practical model system for studying vertebrate retinogenesis^5^. Chicken embryo development is rapid, completing its entire program from blastula to hatchling in 21 days^6^. Recent genomic efforts to improve the quality of the chicken genome assembly combined with newly developed molecular tools for genetic manipulation of this model system have contributed to a renaissance of using the chicken embryo as a robust model to study retinal development^5^. During chick development, the immature E8 retina is composed of multipotent precursor cells, which begin to terminally differentiate into specialized retinal neurons in subsequent days of development. By E18, the retina is nearly mature with photoreceptor (PR), bipolar, amacrine, horizontal, and ganglion cell neurons as well as Muller glial cells having differentiated from these multipotent precursors (Fig. 1)^5^. Progenitor cells yet to exit the cell cycle, as well as each of these specialized retinal cell types are known to express developmental and cell type-specific genes^7^. RNA-sequencing transcriptome data have recently become available to dissect global changes in gene expression during chick retinal development^8,9^. Currently, however, there are no publicly available data sets characterizing epigenetic modifications of the genome in the developing chicken retina. Epigenome studies will complement transcriptome data and contribute to a deeper understanding of vertebrate retinal development.

**Figure 2:**
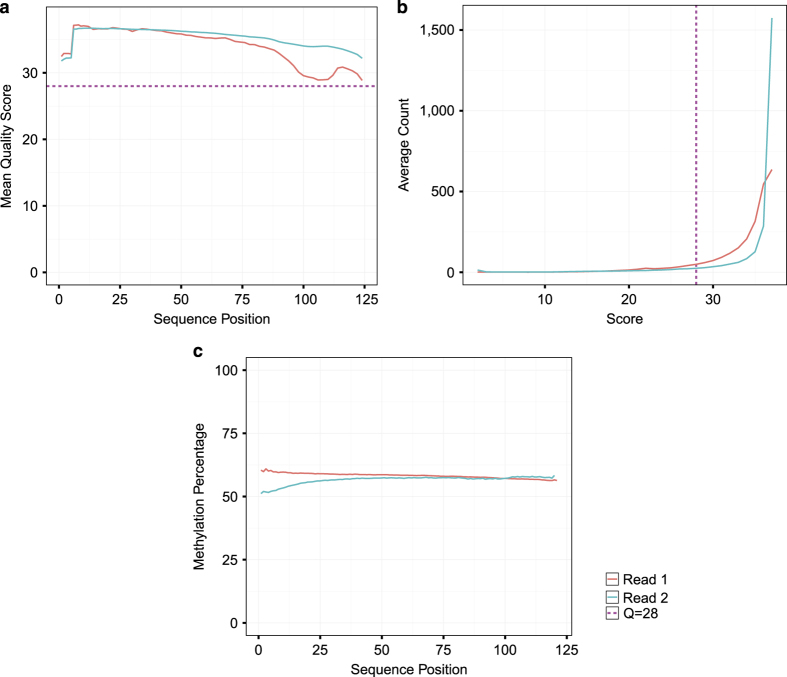
Quality Assessment of FASTQ sequencing data for 125 bp paired end reads. Representative plots showing (**a**) Per Cycle Sequence PHRED score for read 1 and 2, (**b**) Sequence Score Distribution across lanes for raw FASTQ files, and (**c**) per base CpG context methylation percentage.

**Figure 3:**
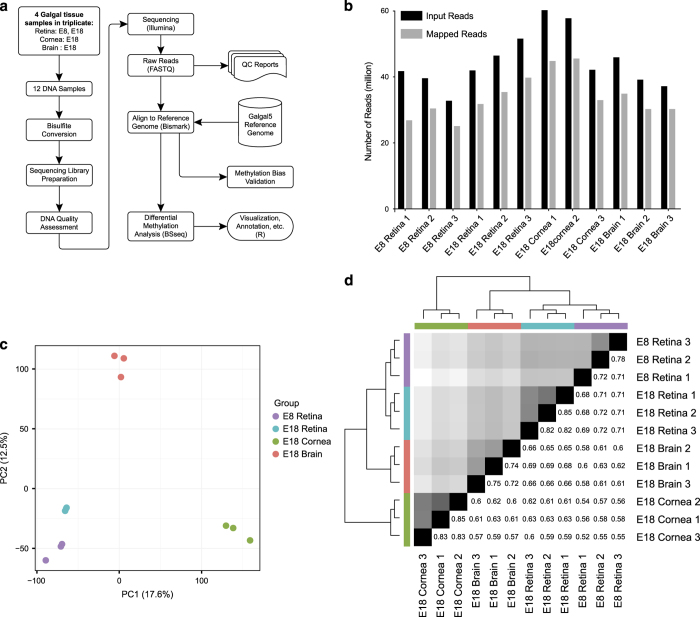
Experimental overview and assessment of read mapping, read length and sample variance. (**a**) Flowchart of the WGBS-seq experiment and data analysis. (**b**) Total number of raw reads compared to number of mapped reads listed per sample. Additional details about the alignment are listed in the table below. (**c**) Principal Component Analysis (PCA) Biplot of samples. Largest source of variation is tissue type, as represented by both PC1 and PC2. (**d**) Hierarchical clustering analyses performed using LOESS smoothed WGBS-seq data. Color code refers to the distance metric used for clustering with white being the lowest correlation value and black being the largest correlation value.

The focus of this project is to characterize global patterns of DNA methylation, an epigenetic modifier of vertebrate genomes, in the developing chicken retina as well as in non-retinal tissues using whole genome bisulfite sequencing (WGBS) NGS technology. These experiments will be critical for downstream characterization of developmental and cell-specific epigenetic regulatory mechanisms during retinal development. The developmental points chosen for analysis were E8 (Fig. 1a–c) and E18 (Fig. 1d–f), which provides epigenetic information for early and late retinal development respectively. E18 whole cornea (Fig. 1e) and brain (not shown) were also included in this analysis as non-retinal ocular and non-retinal neuronal reference tissues respectively. These analyses were conducted using Illumina WGBS in tandem with a standard bioinformatics pipeline to ensure quality of the raw and trimmed sequencing data (Fig. 2) as well as a customized bioinformatics pipeline for robust eukaryotic DNA methylome analysis (Fig. 3).

## Methods

### Embryos

All embryo experiments were conducted with the approval of the James Madison University Institutional Animal Care and Use Committee and in accordance with the National Institutes of Health guide for the care and use of laboratory animals. Fertilized pathogen free commercial Cobb/Hubbard F1 hybrid eggs were obtained from George’s Hatchery (Harrisonburg, VA) and incubated in a rocking chamber held at 38 °C and 50–60% humidity until specified incubation days.

### Tissue processing, histology & imaging:

Chicken embryos were harvested and euthanized at specified days incubated as previously described^9^. Briefly, embryos were decapitated and eyes were enucleated. Whole embryos and whole eyes were imaged prior to eyecups preparation. Isolated corneas and whole brain extracts were saved for subsequent DNA extraction. Histological preparation of eyecups was conducted as previously described^9^. Briefly, eyecups were fixed in 4% paraformaldehyde in 1× PBS for 25 min on ice, equilibrated in 25% sucrose in 1× PBS, and transferred into a 2:1 mixture of 25% sucrose:OCT compound (Electron Microscopy Sciences) on ice for 30 min prior to flash freezing in the same solution in Tissue-Tek Cryomold (Sakura Finetek) and stored in −80 °C. 10 μm thick frozen serial sections were prepared using a CM3050 S Research Cryostat (Leica) with the object and chamber temperatures set to −22 °C and −28 °C respectively. Frozen sections were thawed, H&E stained, and imaged using an EclipseTE2000 inverted microscope (Nikon) and processed with NIS Elements software (Nikon). For retinal dissection, eyecups were incubated for 20 min in HBSS modified media without calcium or magnesium (HBSS -Ca,-Mg;HyClone) at 37 °C to dissociate the retinal pigment epithelium (RPE) layer from the outermost layer of the retina. Retinas were then isolated by tearing away the sclera and gently peeling away the RPE layer. Isolated retinas and corneas were briefly rinsed in cold HBSS -Ca, -Mg. Retinas were immediately transferred to RLT+ lysis buffer (Qiagen; AllPrep kit) containing 2-Mercaptoethanol (Sigma) and vortexed vigorously to dissociate the tissue. Corneas and brain were separately flash frozen and ground into a fine powder using a mortar and pestle prior to being transferred to RLT+/BME lysis buffer solution and vortexed. Samples were stored long term in lysis buffer at −80 °C.

### Genomic DNA isolation

Genomic DNA was collected from twelve embryonic chicken tissues (Table 1). Whole retinas were harvested from E8 (Fig. 1b) and E18 (Fig. 1h) developing chicken embryos as well as whole corneas from E18 embryos (Fig. 1e) and whole brain collected from E18 embryos (not shown). Triplicate samples were obtained for each time point and genomic DNA was extracted from samples using a Qiagen AllPrep Mini Kit per the manufacturer’s instructions. Isolated DNAs were eluted in TE buffer, validated for quality and quantity using UV spectrophotometry, and stored long term at −80 °C. DNAs with an OD260/280 ratio between 1.75 and 1.85 were deemed high quality.

### DNA preparation, bisulfite conversion, and sequencing

Genomic DNA samples were sheared to 200–300 bp fragments using Bioruptor Pico (Diagenode), with 9 cycles of 30 s on and 30 s off. With these samples, sequencing libraries were prepared using NEBNext Ultra II library preparation kit (New England BioLabs) with bisulfite conversion using EZ DNA Methylation-Lightning kit (Zymo) before PCR amplification of adaptor-tagged libraries. Library fragments were assessed using an Agilent Bioanalyzer to plot distribution of DNA fragment peaks. High quality libraries, having a distribution of DNA fragments centered around 300 bps were used for sequencing analysis using the Illumina HiSeq 2,500 sequencing platform yielding 32.8–60.2 million 125 bp paired end sequence reads per sample (Fig. 3b).

### Quality validation and read mapping:

Between 32.8–60.2 million paired end sequence reads were obtained per sample from the Johns Hopkins School of Medicine Genetic Resource Core Facility. Quality of individual sequences within FASTQ files were evaluated using custom quality control analysis (see Code Availability), including per cycle quality analysis which plots the Phred quality score distribution on the y axis for each cycle of the sequencing by synthesis reaction plotted on the x axis (Fig. 2a, Supplementary Fig. 1) as well as per sequence quality analysis which plots mean Phred quality scores on the x axis against the overall number of reads corresponding to that Phred score on the y axis (Fig. 2b, Supplementary Fig. 2). Figure 2 demonstrates a representative sample FASTQ sequencing files from each tissue used in this analysis. Each FASTQ file had an average per base Phred score > 28 as well as the vast majority of sequencing reads with a mean Phred score > 28, both conventional thresholds denoting high quality base calls.

To correct for bias of methylation percentage on the ends of the reads, several bases were trimmed off prior to subsequent analysis using Trim Galore! (see Code Availability 1). The number of bases trimmed was determined empirically by taking a subset of the reads through the bioinformatics pipeline and observing the methylation bias. In addition, adaptor sequences and bases below the Phred score of 20 from the 3′ end were removed further increasing the average per base Phred score or reads used in downstream analysis (Supplementary Fig. 4). Trimming altogether removed 5.9 to 13% of the sequenced base pairs (Table 2). Using trimmed reads, no significant bias in methylation was observed (Fig. 2c,Supplementary Fig. 3). Figure 3a demonstrates our experimental overview including the bioinformatics pipeline employed following quality validation of sequence reads. High quality sequence reads were aligned to the UCSC Gallus gallus reference genome (galGal5) preprocessed for bisulfite sequencing using bismark software^10^ (See Code Availability 2), yielding a range of 65 to 80% uniquely aligned reads (Fig. 3b and Table 3).

### Methylation data analysis

Aligned bisulfite sequence reads were processed using the Bioconductor package bsseq^11^. Local-likelihood smoothing was performed on the datasets to improve the precision of methylation frequencies. Smoothing parameters were adjusted to fit the downstream analysis, i.e. differentially methylated region finding versus large block finding. Gene and CpG island annotations for *Gallus gallus* v5 chicken genome were obtained from the UCSC genome annotation database, and global methylation was measured for each sample group using bsseq smoothed methylation data (Supplementary Fig. 5). Global methylation showed a dip near the TSS, as is typically expected. In the gene bodies, methylation varied widely, showing little difference to the methylation across the whole genome. Finally, CpG islands were mostly unmethylated, with a median of 66% of CpG islands having an average methylation below 20%.

### Code availability

The following software and versions were used for quality control and data analysis as described in the main text:

1. FastQC, version 0.11.4 was used for quality analysis of raw FASTQ sequencing data: https://www.bioinformatics.babraham.ac.uk/projects/fastqc/
2. Trim Galore!, version 0.4.1 was used for adaptor and end-trimming of raw FASTQ sequencing data: http://www.bioinformatics.babraham.ac.uk/projects/trim_galore/
3. Bismark, version 0.16.3 was used for bisulfite-sequencing-specific alignment of raw FASTQ sequencing data: http://www.bioinformatics.babraham.ac.uk/projects/bismark/

All code used for quality assessment and data analysis in this study is available at: https://github.com/isaclee/chicken

## Data Records

Raw FASTQ files for the whole genome bisulfite-seq libraries were deposited to the NCBI Sequence Read Archive (SRA) (Data Citation 1), and have been assigned BioProject accession PRJNA389197 (Table 1; Data Citation 1).

## Technical Validation

### Quality control-DNA library integrity

Quality of the bisulfite library was assessed using an Agilent Bioanalyzer to plot distribution of DNA fragment peaks. High quality libraries, having a distribution of DNA fragments centered around 300 bps without adaptor/primer dimer peaks, were used for sequencing analysis.

### Bisulfite sequencing data quality

Mean Phred quality scores of the sequenced reads fall in the high quality range, as shown by per base (Fig. 2a, Supplementary Figs 1 and 4) and per sequence (Fig. 2b, Supplementary Fig. 2) quality analysis. 25.0 to 45.5 million reads were mapped to the chicken reference galGal5 genome assembly (Table 3). No significant bias in DNA methylation percentage was observed with respect to the sequence position along the read (Fig. 2c, Supplementary Fig. 3).

### Data reproducibility

Using smoothed methylation values at all available CpG loci, we performed principal component analysis and hierarchical clustering analysis to test the reproducibility of the methylation data (Fig. 3c,d). The resulting within-group and between-group Pearson correlations were calculated to report numerical evidence of the conclusion (Fig. 3d and Supplementary Fig. 6). As expected, each triplicate group shared similar variances in the first two principal components and grouped into distinct clusters. Likewise, highest within-group correlations were observed within the triplicates, and the highest between-group correlation was observed between the two developmental stages of retinal samples.

## Usage Notes

The bioinformatics pipeline applied to our data set outlined in Fig. 3a was achieved using a collection of freely available, open access tools. However, these analyses are interchangeable with many other currently available tools. Our raw FASTQ data can be aligned to any available chicken reference genome, including the most recent 2015 galGal5 assembly, using a variety of freely available bisulfite-converted sequence aligners. In this study we used the Bismark methylation aligner^10^, however, we expect that similar alignment results can be achieved using the bsmooth pipeline^11^. Alignment of the FASTQ data in the form of bam files can be viewed using popular genome browsers such as the UCSC Genome Browser^12^, the Ensembl Browser^13^, or the Broad Institute’s Integrative Genome View^14^. Subsequent differential methylation analysis using these data can be carried out using the R/bioconductor package bsseq^11,15^ or other publicly available packages such as methylkit^16^ may also be used for this analysis.

Our data set will be useful for a variety of studies investigating developmental and tissue-specific changes in DNA methylation in the vertebrate retina. There are, however, several considerations that must be taken into account when using these data for downstream analysis. First, DNAs were extracted from whole retina, whole brain, and whole cornea without any enrichment for cell type. Therefore, resulting DNA methylation patterns are representative of heterogeneous mixtures of different cell types within these tissues. Second, the quantity of sequenced and mapped reads per sample in this study (Fig. 3b) is sufficient for robust differentially methylated region and block analysis, but is below the suggested threshold for small or single nucleotide methylation analysis. We chose this coverage to maximize the number of biological replicates, following work by Ziller *et al.* who demonstrated that 5X-15X coverage was sufficient^17^. Finally, due to the decreased nucleotide complexity after bisulfite conversion, the alignment of the sequence reads and hence the methylation measurements may vary depending on the reference genome used for alignment^18^. Taking these considerations into account, these data will be a useful resource for the vision research community to thoroughly investigate critical changes in DNA methylation that take place during the complex process of vertebrate retinal development as well as differences in DNA methylation between other ocular and neuronal tissues. These data can also be used in conjunction with transcriptome data to characterize epigenetically regulated transcriptional networks critical for retinal development.

## Additional Information

**How to cite this article:** Lee, I. *et al.* Whole genome DNA methylation sequencing of the chicken retina, cornea and brain. *Sci. Data* 4:170148 doi: 10.1038/sdata.2017.148 (2017).

**Publisher’s note:** Springer Nature remains neutral with regard to jurisdictional claims in published maps and institutional affiliations.

## Supplementary Material



Supplementary Figures

## Figures and Tables

**Table 1:** BS-seq profiling to evaluate developmental and tissue-specific retinal DNA methylation.

**Subject**	**Source**	**Sample Name**	**Method 1**	**Method 2**	**Data Accession**
Chicken 1	embryonic day 8 retina	E8 retina 1	DNA extraction	WGBS-seq	SAMN07191783
Chicken 2	embryonic day 8 retina	E8 retina 2	DNA extraction	WGBS-seq	SAMN07191783
Chicken 3	embryonic day 8 retina	E8 retina 3	DNA extraction	WGBS-seq	SAMN07191783
Chicken 1	embryonic day 18 retina	E18 retina 1	DNA extraction	WGBS-seq	SAMN07191784
Chicken 2	embryonic day 18 retina	E18 retina 2	DNA extraction	WGBS-seq	SAMN07191784
Chicken 3	embryonic day 18 retina	E18 retina 3	DNA extraction	WGBS-seq	SAMN07191784
Chicken 1	embryonic day 18 cornea	E18 cornea 1	DNA extraction	WGBS-seq	SAMN07191785
Chicken 2	embryonic day 18 cornea	E18 cornea 2	DNA extraction	WGBS-seq	SAMN07191785
Chicken 3	embryonic day 18 cornea	E18 cornea 3	DNA extraction	WGBS-seq	SAMN07191785
Chicken 1	embryonic day 18 brain	E18 brain 1	DNA extraction	WGBS-seq	SAMN07191786
Chicken 2	embryonic day 18 brain	E18 brain 2	DNA extraction	WGBS-seq	SAMN07191786
Chicken 3	embryonic day 18 brain	E18 brain 3	DNA extraction	WGBS-seq	SAMN07191786

**Table 2:** BS-seq read and filtering statistics.

**Sample Name**	**Sequencer**	**Total basepairs (Gb)**	**Basepairs passing filters (Gb)**	**Retained basepairs (%)**
E8 retina 1	Illumina HiSeq 2,500	9.9	9.12	92.1
E8 retina 2	Illumina HiSeq 2,500	15.06	13.87	92.1
E8 retina 3	Illumina HiSeq 2,500	14.45	13.42	92.9
E18 retina 1	Illumina HiSeq 2,500	10.54	9.75	92.5
E18 retina 2	Illumina HiSeq 2,500	10.44	9.08	87
E18 retina 3	Illumina HiSeq 2,500	11.49	10.82	94.1
E18 cornea 1	Illumina HiSeq 2,500	12.9	11.94	92.5
E18 cornea 2	Illumina HiSeq 2,500	8.19	7.68	93.8
E18 cornea 3	Illumina HiSeq 2,500	11.62	10.55	90.8
E18 brain 1	Illumina HiSeq 2,500	9.79	8.95	91.5
E18 brain 2	Illumina HiSeq 2,500	9.3	8.47	91.1
E18 brain 3	Illumina HiSeq 2,500	10.49	9.78	93.2

**Table 3:** BS-seq mapping statistics.

**Sample Name**	**Aligner**	**Read Length (bp)**	**Million read-pairs**	**Uniquely mapped reads (%)**
E8 retina 1	Bismark v.0.16.3	2×125	41.75	64.2
E8 retina 2	Bismark v.0.16.3	2×125	39.59	76.8
E8 retina 3	Bismark v.0.16.3	2×125	32.76	76.6
E18 retina 1	Bismark v.0.16.3	2×125	41.97	75.8
E18 retina 2	Bismark v.0.16.3	2×125	46.47	76.2
E18 retina 3	Bismark v.0.16.3	2×125	51.6	77.1
E18 cornea 1	Bismark v.0.16.3	2×125	60.24	74.4
E18 cornea 2	Bismark v.0.16.3	2×125	57.79	78.8
E18 cornea 3	Bismark v.0.16.3	2×125	42.16	78.2
E18 brain 1	Bismark v.0.16.3	2×125	45.96	76
E18 brain 2	Bismark v.0.16.3	2×125	39.16	77.3
E18 brain 3	Bismark v.0.16.3	2×125	37.19	79.4
