# Supplementary material for: Whole genome DNA methylation sequencing of the chicken retina, cornea and brain
Source: Sci Data. 2017 Oct 10;4:170148. doi: 10.1038/sdata.2017.148 (PMC5634322; doi:10.1038/sdata.2017.148)
Supplement: Supplementary Figures [file sdata2017148-s2.pdf]

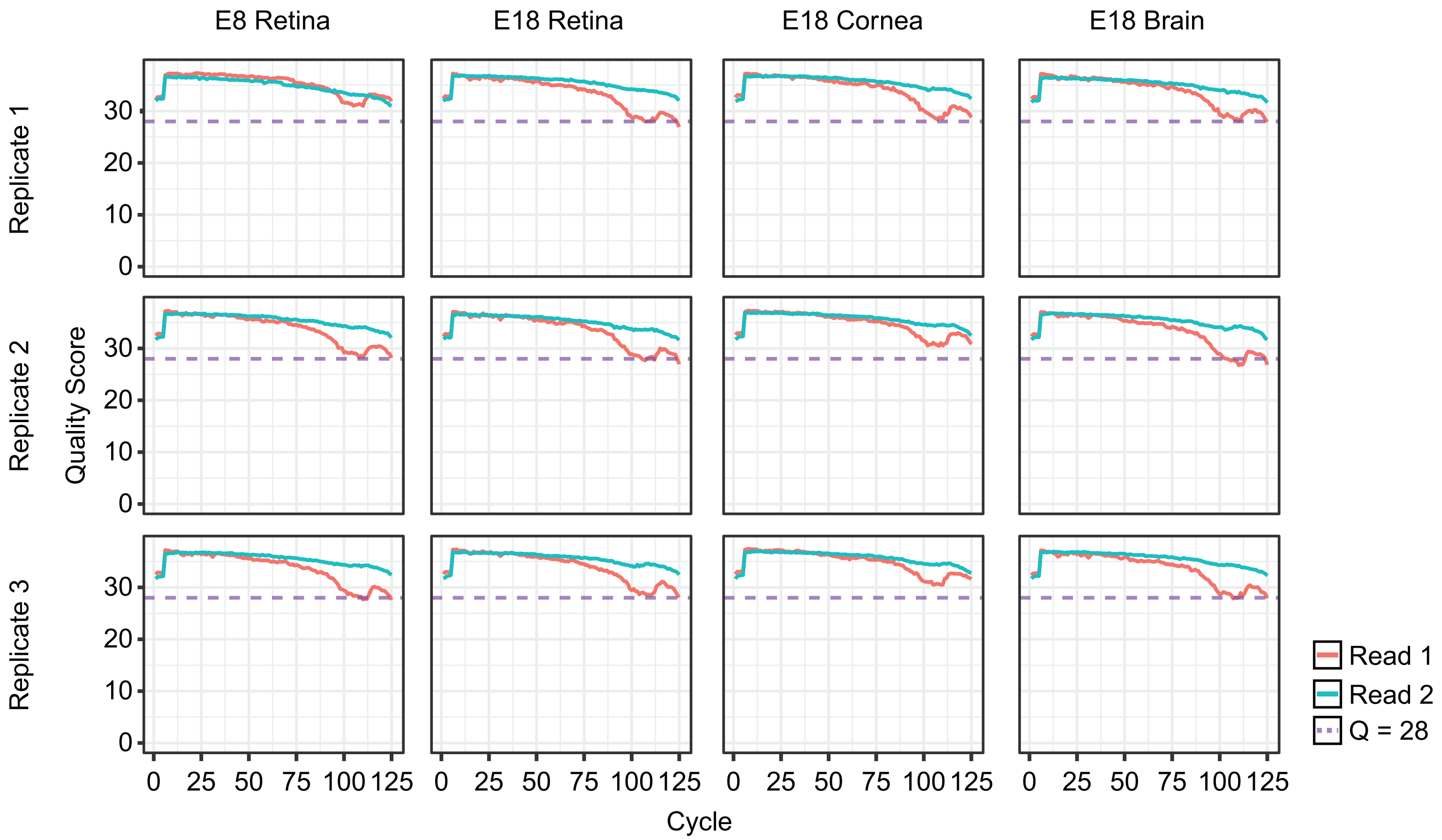

Supplementary figure 1. Per Cycle Sequence PHRED score of the raw fastq sequences. For every sample, the average score is >28.

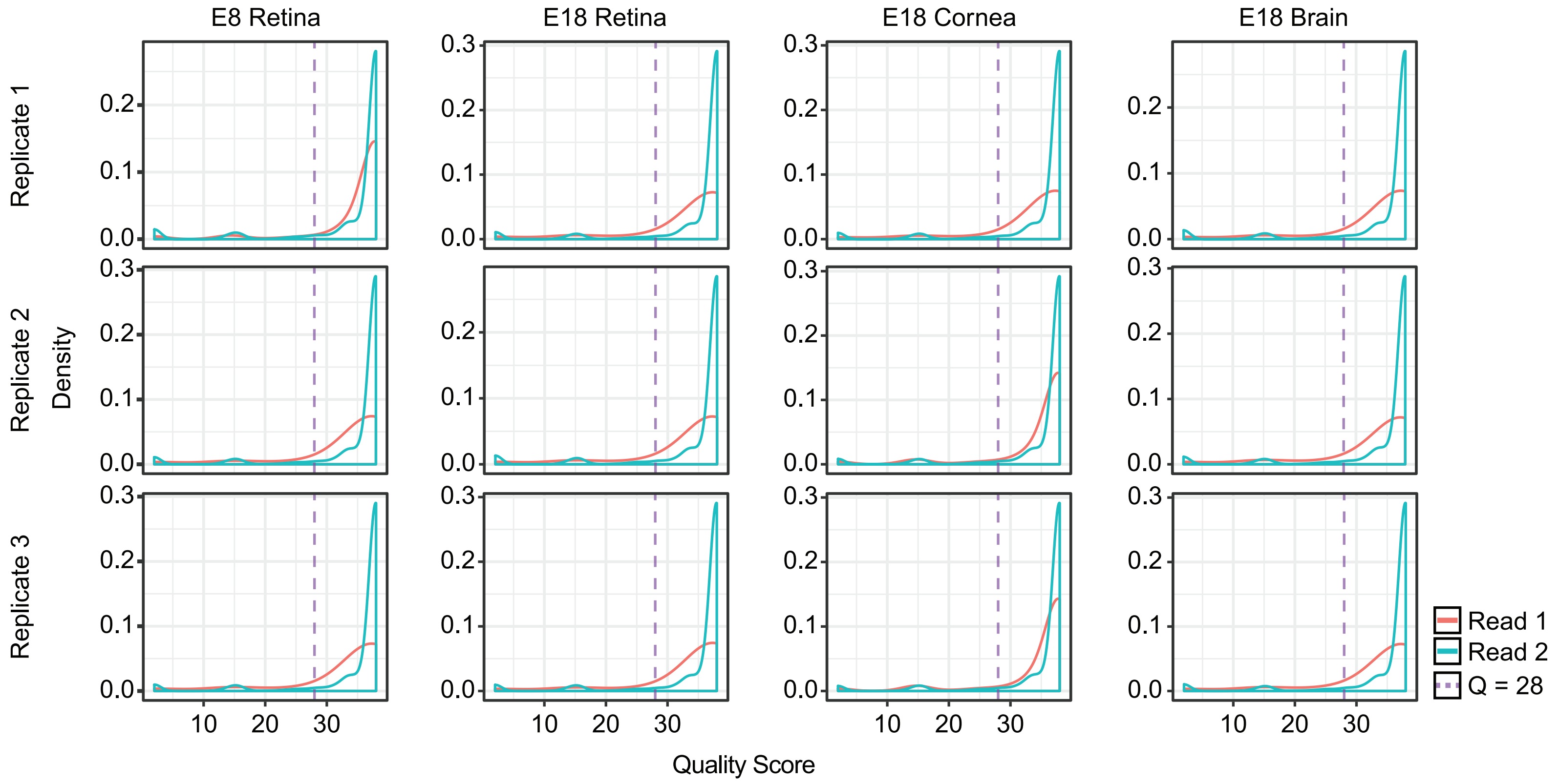

Supplementary figure 2. Density plots of PHRED Score distribution of the raw fastq sequences. Vast majority of sequences have a score of > 28.

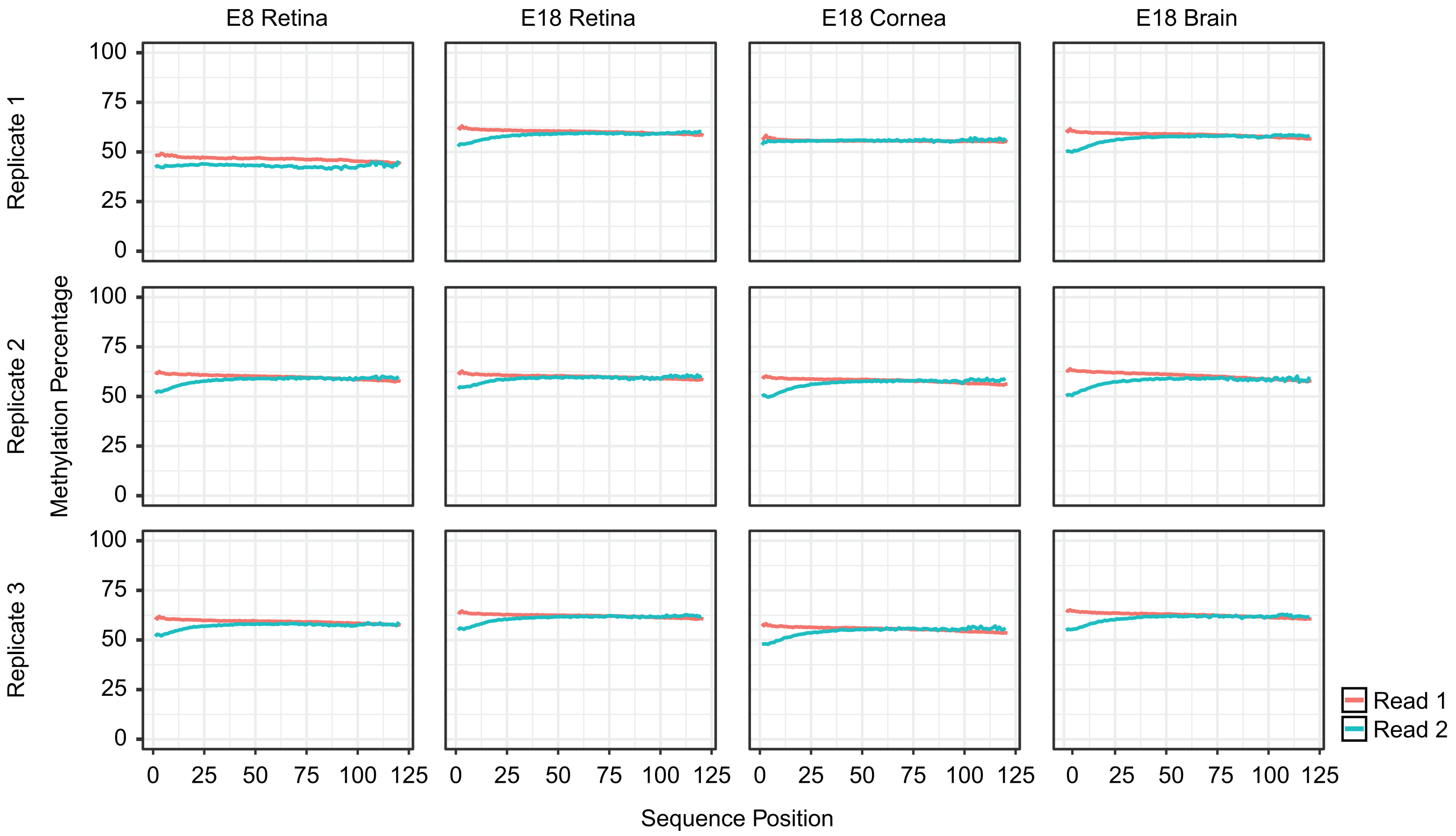

Supplementary figure 3. Per base CpG context methylation percentage across all samples following trimming.

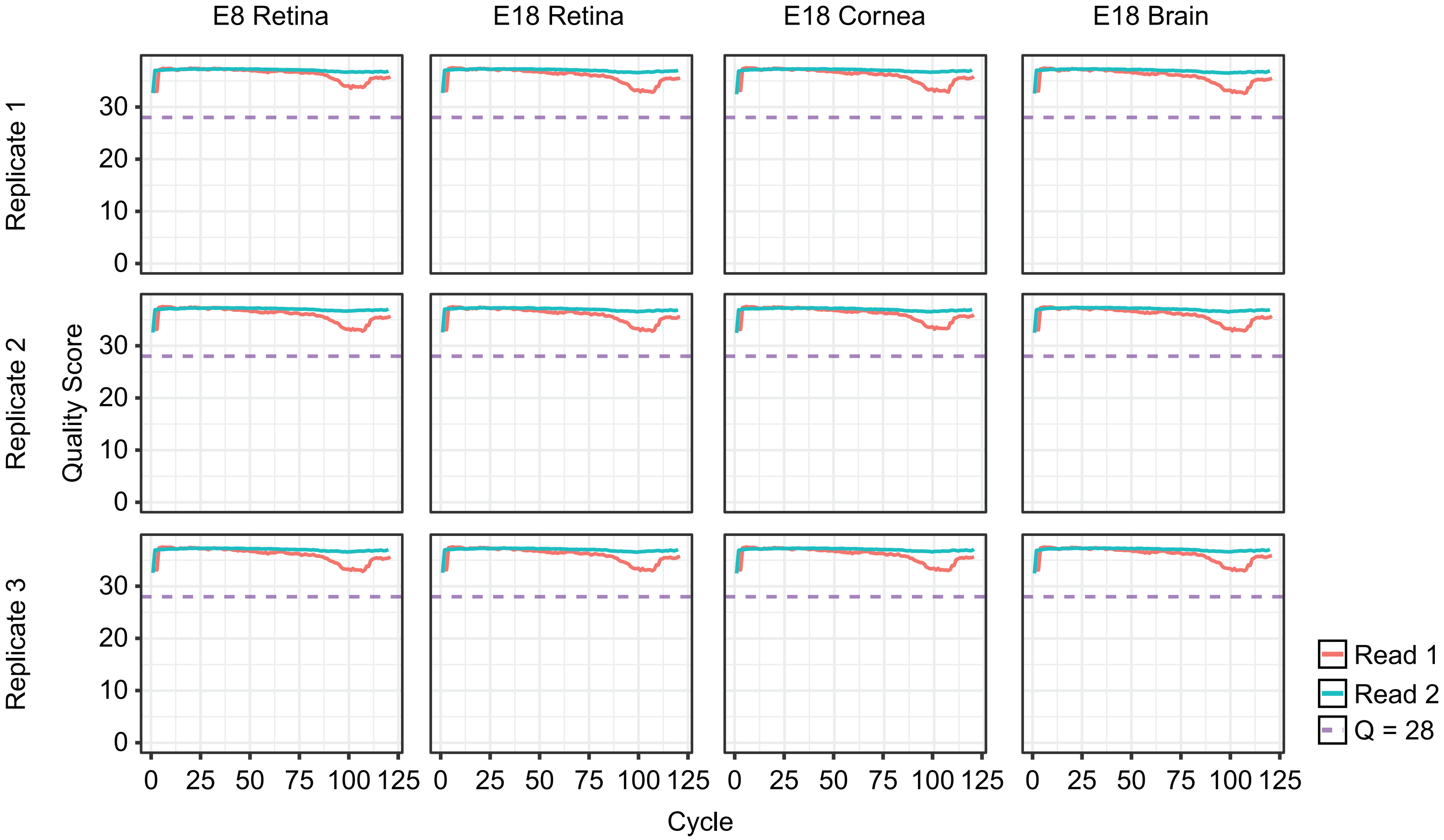

Supplementary figure 4. Per Cycle Sequence PHRED score of the aligned sequences.

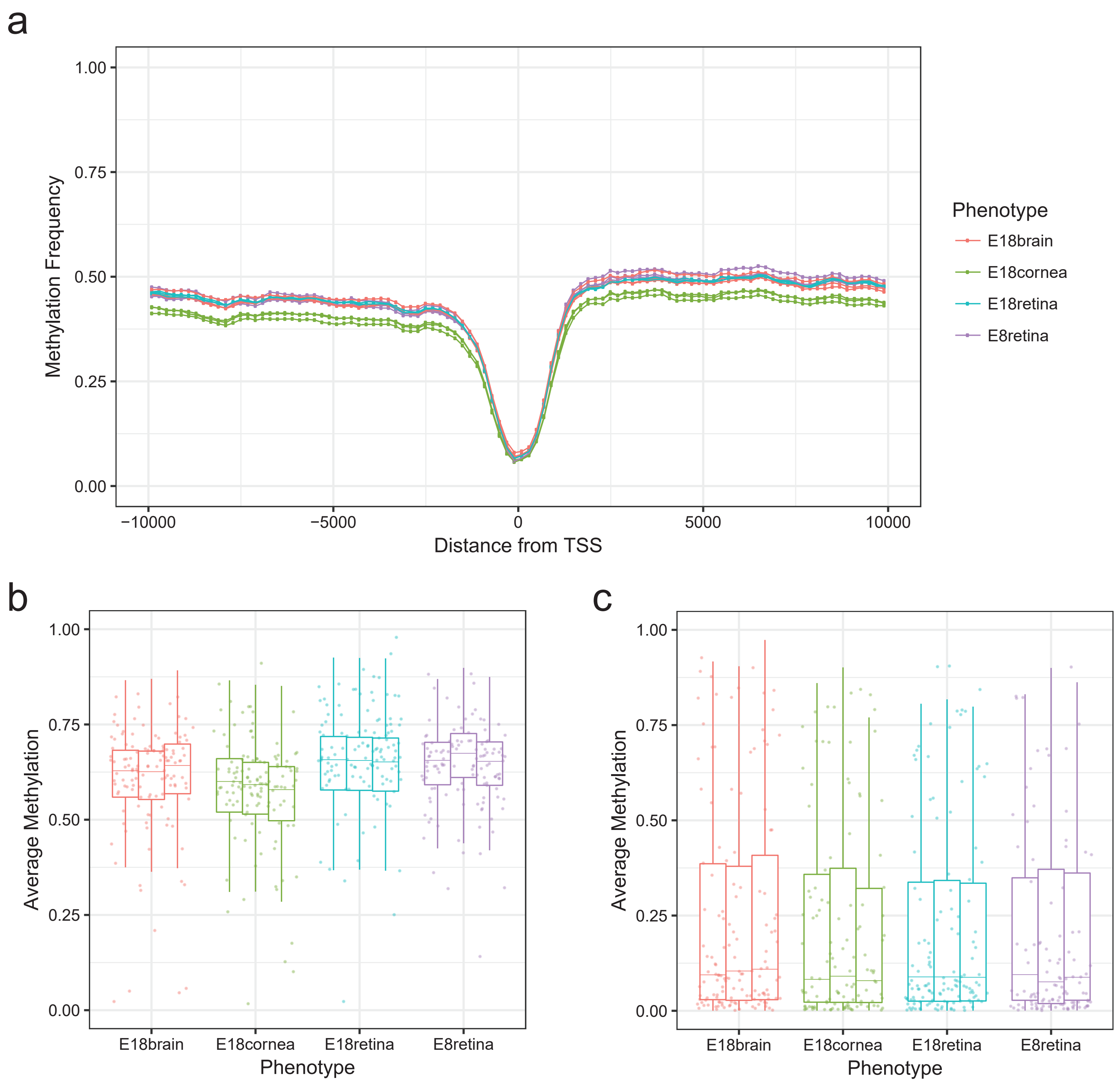

Supplementary figure 5. Assessment of global methylation in annotated regions. a) Methylation frequency 10kb upstream (denoted by the - sign) and downstream (+) of transcription start sites, b) box plots of average methylation frequencies in gene bodies, and c) box plots of average methylation frequencies in CpG islands.

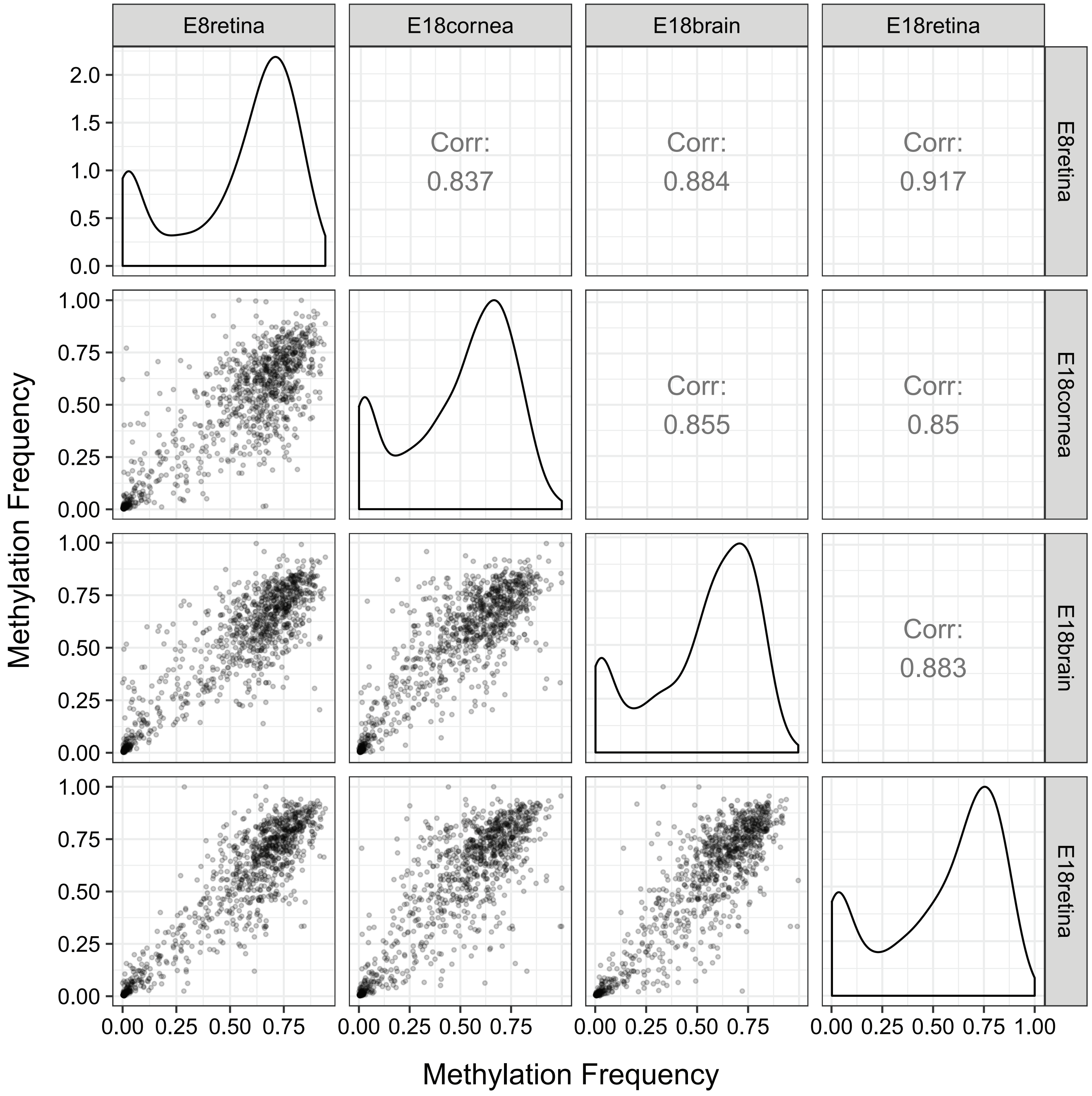

Supplementary figure 6. Assessment of correlation between subsets of sample groups. Bottom left shows scatter plots of methylation frequency across the genome between pairs of groups, top right shows Pearson correlation values for the pairs, and diagonal shows the density distribution of methylation frequencies for each group.
